# Peripheral blood derived gene panels predict response to infliximab in rheumatoid arthritis and Crohn's disease

**DOI:** 10.1186/gm463

**Published:** 2013-06-28

**Authors:** Bertalan Mesko, Szilard Poliska, Andrea Váncsa, Zoltan Szekanecz, Karoly Palatka, Zsolt Hollo, Attila Horvath, Laszlo Steiner, Gabor Zahuczky, Janos Podani, and Laszlo Nagy

**Affiliations:** 1 Department of Biochemistry and Molecular Biology, Debrecen, Egyetemtér, 4028, Hungary; 2 MTA-DE "Lendulet" Immunogenomics Research Group, Research Center for Molecular Medicine, University of Debrecen, Medical and Health Science Center Debrecen,Egyetemtér, 4028, Hungary; 3 Center for Clinical Genomics and Personalized Medicine, Medical and Health Science Center, University of Debrecen, Debrecen, Egyetemtér, 4028, Hungary; 4 Department of Rheumatology, Institute of Medicine, University of Debrecen, Medical and Health Science Center, Debrecen, Egyetemtér, 4028, Hungary; 5 2nd Department of Internal Medicine, University of Debrecen, Medical and Health Science Center, Debrecen, Egyetemtér, 4028, Hungary; 6 EGIS Pharmaceuticals, H-1106 Budapest, Keresztúriút 30-38, Hungary; 7 UD-GenoMed, Ltd., Debrecen, Egyetemtér, 4012, Pf 52, Hungary; 8 Biological Institute, LorandEötvös University, H-1117 Budapest, Egyetemtér, Hungary

## Abstract

**Background:**

Biological therapies have been introduced for the treatment of chronic inflammatory diseases including rheumatoid arthritis (RA) and Crohn's disease (CD). The efficacy of biologics differs from patient to patient. Moreover these therapies are rather expensive, therefore treatment of primary non-responders should be avoided.

**Method:**

We addressed this issue by combining gene expression profiling and biostatistical approaches. We performed peripheral blood global gene expression profiling in order to filter the genome for target genes in cohorts of 20 CD and 19 RA patients. Then RT-quantitative PCR validation was performed, followed by multivariate analyses of genes in independent cohorts of 20 CD and 15 RA patients, in order to identify sets ofinterrelated genes that can separate responders from non-responders to the humanized chimeric anti-TNFalpha antibody infliximab at baseline.

**Results:**

Gene panels separating responders from non-responders were identified using leave-one-out cross-validation test, and a pool of genes that should be tested on larger cohorts was created in both conditions.

**Conclusions:**

Our data show that peripheral blood gene expression profiles are suitable for determining gene panels with high discriminatory power to differentiate responders from non-responders in infliximab therapy at baseline in CD and RA, which could be cross-validated successfully. Biostatistical analysis of peripheral blood gene expression data leads to the identification of gene panels that can help predict responsiveness of therapy and support the clinical decision-making process.

## Background

Biological therapies targeting tumor necrosis factor alpha (TNFα) have been introduced for the treatment of chronic inflammatory diseases including rheumatoid arthritis (RA) and Crohn's disease (CD). Up tothe end of 2010 [1], more than two million patients worldwide had received treatment with anti-TNFα biologic agents, such as infliximab, adalimumab and etanerceptforconditions such as RA and CD. The efficacy of these biologics differs from patient to patient and these agents are rather expensive, sothey should not be used to treat primary non-respondersin the long term. In this study, we aimed at predicting patient response to infliximab, ahumanized chimeric anti-TNFα antibody, from the genomic perspective. Infliximab is a genetically constructed immunoglobulin G1 murine-humanchimeric monoclonal antibody binding both to thesoluble subunit and the membrane-bound precursor of TNFα; and has proven to be anefficacious treatment for both RA[2] and CD [3].

RA and CD are believed to have a common pathogenetic background because they can be associated with overlapping biological processes, includingchanged inflammatory response[4], therefore it is reasonable to expect that overlapping gene panels could predict the response to the same therapy in these two conditions.

Gene expression profiling has been successfully used on tissue samples or blood for the identification of biomarkers and/or genome classifiers in various disorders, such as breast cancer [5] and asthma [6]. Peripheral blood mononuclear cells (PBMCs) contain cells affected by inflammation, such as circulating monocytes, T-lymphocytesand B-lymphocytes. Therefore gene expression patternsof PBMCsmay reflect mechanisms of the disease, and the challenge is to producepharmacogenomics biomarkers and/or genome classifiersfor clinical decision making through the development of assays based on gene panels predicting response to therapies or disease progression [7]. It would be particularly interesting to know how the PBMCs of patients with CD or RA respond to the same biological therapy and, if discriminating gene panels are available, what the degree of similarity between such panels is in predicting the outcome of therapy and disease progression.

There is a clear need for a set of biomarkers and/or genome classifiers predicting response to infliximab therapy, underscored by two important problems. First, about 35% of patients with CD [8] and 20% to 40% of patients with RA [9,10] fail to respond to this therapy. Second, efficacy may decline afterswitching to a second TNFα inhibitor[11]. Therefore, predicting whether a patient will respond to a particular therapy before starting the first therapeutic option is clearly an unmet medical need. This predictive ability would have a strong effect on the use of these medications, and could lower healthcare costs and give the patient the opportunity to receive 'personalized' therapy. Biomarkers or sets of biomarkers and/or genome classifiers predicting response to therapy by using the least invasive peripheral blood sampling have clear advantages [12].

The response to infliximab therapy has been examined in CD by using colon biopsy samples [13] and in RA using blood [14,15] as well as synovial biopsy [16]. A comparison of the response from the genomic perspective in both conditions in PBMCs has never been documented.

In the present study, we performed PBMC global gene expression profiling for filtering the genome for target genes on one cohort of patients with CD and one cohort with RA. Wethen performed RT-quantitative PCR gene expression in PBMCs on independent cohorts,followed by multivariate analyses to identify interrelated gene sets that can differentiate responders from non-responders to infliximab therapy in an independent cohort. Compared to studies in which single genes differentiating between responders and non-responders are the focus, our analysis put an emphasis on identifying interrelated gene panels showing differences between the above-mentioned groups.

Our results demonstrate that peripheral blood gene expression profiles are suitable for determining panels of interrelated genes with high discriminatory power,as shown by cross-validation analyses that can differentiate responders from non-responders to infliximab therapy at baseline in cohorts of patients with CD and with RA. We found that distinct, non-overlapping panels of interrelated genes can be used to predict the responder status in these conditions.

## Methods

### Patient samples

The Institutional Review Board of University of Debrecen Medical and Health Science Center approved the clinical protocol and study, which were in compliance with the Helsinki Declaration. Signed informed consent was obtained from all individuals providing blood sample.

**Figure 1:**
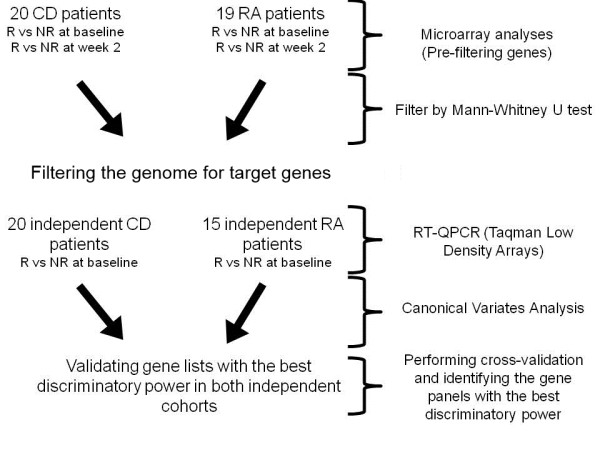
**Schematic outline of the study design**. CD,Crohn's disease;NR, non-responder; R, responder; RA, rheumatoid arthritis.

In total, 40 Caucasian patients with CD (16 females, 24 males) diagnosed by clinicians; and 34 Caucasian patients (28 females, 6 males) who met the 2010 European League Against Rheumatism/American College of Rheumatology(ACR) classification criteria[17] for RA were included in the study; all of whom had active disease at the time blood was drawn. Regarding the study design, 20 patients with CD and 19 with RA were included in the first test cohort for microarray experimentsampling at baseline and week 2. For the validation cohort, samples from 20patients with CDand 15with RAat baseline were included in the RT-quantitative PCR experiments. The schematic outline of our study design is given in Figure 1.

All blood samples were obtained after the participantsfasted overnightfor 12 hours locally between 8:00 am and 9:00 ambefore the first admission of infliximab at week 0 (baseline) and the second at week 2; and were processed within one hour after sample collection.

Medicationsthatremained unchanged during the study and patient co-morbiditiesare shown in Tables 1 and 2. Co-medication was given after blood was taken.

**Table 1:** Summary of the clinical parameters of patients with Crohn's disease

	Responders	Non-responders	Responders	Non-responders	
	Test cohort		Validation cohort		Difference
**At baseline **	14	6	13	7	
Gender (male/female)	8/6	4/3	8/5	4/4	
Age (years)	36.2±14.6	36±15.4	26.8±8.7	30.2±5.1	Non-significant
CDAI	319.6±41.3	351.5±33.1	338.3±61.7	370.7±48.1	Non-significant
CRP (mg/ml)	22.7±20.2	13.5±28.9	27.2±36.2	16.5±11.6	Non-significant
Hemoglobin (g/l)	125.1±17.6	120.6±27.6	130±17.9	127±12	Non-significant
Leukocytes(g/l)	9±3.3	8±3.1	7.5±2.3	9.2±2.8	Non-significant
Neutrophils (%)	70±8.9	74.5±8.9	74±8.1	72.8±5.7	Non-significant

CDAI, Crohn's disease activity index; CRP, C-reactive protein. Data are shown as mean ±SD.

**Table 2:** Summary of the clinical parameters of patients with rheumatoid arthritis

	Responders	Non- or moderate responders	Responders	Non- or moderate responders	
	Test cohort		Validation cohort		Difference
**At baseline **	6	13	4	11	
Gender (male/female)	1/5	2/11	0/4	3/8	
Age (years)	44.3±9.3	47±10.7	54±17	56.2±6.8	Non-significant
DAS28	5.6±0.3	5.2±0.7	5.2±0.06	5.4±0.5	Non-significant
HAQ	1.2±0.7	2±0.6	1.5±1.1	1.9±0.7	Non-significant
CRP (mg/ml)	16.8±18.3	28.3±23.8	18.9±18.3	9.5±10.3	Non-significant
DMARDs	2.8±0.9	2.6±0.7	3±0.8	2.7±1.4	Non-significant

CRP, C-reactive protein;DAS28, Disease Activity Score; HAQ, Health Assessment Questionnaire; DMARD, disease-modifying anti-rheumatic drug (methotrexate). Data are shown as mean±SD.

Clinical parameters, including Crohn's disease activity score (CDAI), C-reactive protein (CRP), hemoglobin, leukocyte and neutrophil counts in CD; and Disease Activity Score (DAS28), Health Assessment Questionnaire (HAQ), CRP and disease-modifying anti-rheumatic drugs (DMARDs) in RA were assessed at the time of the first infliximab infusion (baseline), at the second infusion (week 2), and at week 6 or 14 when the responder statuswas determined based on clinicians' assessment.

The inclusion criteria in RA were fulfillment of the 2010 European League Against Rheumatism/ACR classification criteria; age between 20 and 60 years; failure to respond to at least two DMARDs; active disease (DAS28 >3.2);and anti-TNFα therapy-naive patients or previous anti-TNFα use at least 3 months prior to blood sampling.Prednisone therapy ≤10 mg per day was allowed provided that the dosage had been stable for at least 2 months before entryand non-steroidal anti-inflammatory drugs were allowed in doses stable for at least 1 month before baseline.Patients were on maximal-tolerable methotrexate treatment (5 to 30 mg per week), which had to be stable for at least 4 weeks before baseline.Exclusion criteria were pregnancy or breastfeeding; current or recent cancer; active infectious disease; a history of an acute inflammatory joint disease of different origin; and smoking.

Inclusion criteria in CD were clinically diagnosed CD; age between 20 and 60 years; CDAI >250; anti-TNFα therapy-naive patients; and prednisolone therapy with a dosage of less than 10 mg/day.Exclusion criteria were pregnancy or breastfeeding; current or recent cancer; active infectious disease; and smoking.

Responder status was determined by a CDAI decrease of 100 points compared to baseline in CD at week 6; and by ACR categories at week 14 in RA (ACR0% and ACR20% improvement represent the non-responder; ACR50% and ACR70% represent the responderstatus).

### Peripheral blood mononuclear cell and RNA isolation

Venous peripheral blood samples were collected (10 ml) in Venous Blood Vacuum Collection Tubes containing EDTA (BD Vacutainer K2EDTA, Becton Dickinson, New Jersey, United States). PBMCs were separated by Ficoll gradient centrifugation. Total RNA was extracted from PBMCs using Trizol reagent (Invitrogen, Carlsbad, California, United States), according to the manufacturer's protocol. RNA quality was checked onan Agilent Bioanalyzer 2100 (Agilent Technologies, Santa Clara, California, United States); all samples had a 28S/18S ratio between 1.5 and 2.0 and the RNA Integrity Number was between 9 and 10. Quantity was determined usingNanoDrop (Thermo Scientific, Waltham, Massachusetts, United States).

### Microarray

AffymetrixGeneChip Human Gene 1.0 ST array (Affymetrix, Santa Clara, California, United States) was used to analyzethe global expression pattern of 28,869 well-annotated genes. Ambion WT Expression Kit (Applied Biosystems, Foster City, California, United States) and GeneChip WT Terminal Labeling and Control Kit (Affymetrix) were used for amplifying and labeling 250 ng of RNA samples. Samples were hybridized at 45°C for 16 hours and then standard washing protocol was performed using GeneChip Fluidics Station 450 and the arrays were scanned on GeneChip Scanner 7G (Affymetrix).

### Univariate data analysis

Microarray data (Gene Expression Omnibus accession number: [GEO:42296]) were analyzed with Genespring GX10 (Agilent Technologies). Affymetrix data files were imported using the robust multi-array analysis algorithm and median normalization was performed, then genes with low expression levels were filtered out removing the lowest 20% based on raw intensity values.Differentially expressed genes between responders versus non-responders and baseline versus week 2 conditions were identified using Mann-Whitney U test with a fold-change cut off 1.5.

### RT-quantitative PCR measurements

Gene expression data were obtained using TaqMan LowDensity Array (Life Technologies, Carlsbad, California, United States). Our custom-designed TaqMan LowDensity Array card allows for two samples to be run in parallel against 96 TaqMan gene expression assays. Based on our microarray experiment and the relevant literature, 91 genes were chosen; the remaining fivegenes were housekeeping genes for normalization (*ACTB*, *GAPDH*, *HPRT1*, *PPIA *and*RPLP0*). cDNA was generated with High Capacity cDNA Reverse Transcription Kit (Life Technologies) according to manufacturer's protocol. RT-quantitative PCR amplification was performed using an ABI Prism 7900HT instrument (Life Technologies).Relative gene expression levels were calculated by a comparative Ct method that results in normalizing to PPIA(PeptidylprolylIsomerase A (Cyclophilin A))expression for each sample.

### Multivariate data analysis: canonical variates analysis or linear discriminant analysis

Separation between predefined groups of objects is best revealed by canonical variates analysis (CVA). This method is the extension of linear discriminant analysis (LDA), and the two terms are used equivalently in this study. CVA was used to determine whether the groups of respondersand non-responders are separable in the multidimensional space spanned by the genetic variables, and if so, which gene subsets have the best discriminatory power. The results of CVA are the so-called canonical scores obtained from the canonical functions derived through eigenanalysis, which serve as coordinates of observations in the canonical space. A more detailed description about CVA is included in Additional file 1.

A receiver operating characteristic (ROC) curve shows the performance of a binary classifier. The curve provides a complete sensitivity and specificity report, where each point represents a sensitivity-specificity pair at various cut points. The area under the ROC curve (AUC) is a diagnostic measure indicating how a parameter can distinguish between two groups (responder and non-responder).

### Automated gene panel generation

**Figure 2:**
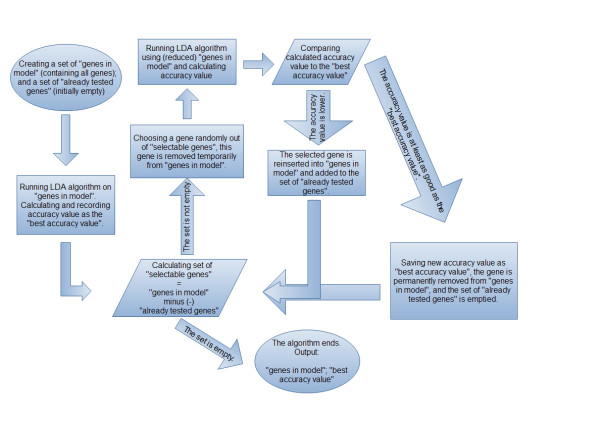
**Schematic flowchart of automatic gene panel generation**. LDA, linear discriminant analysis.

LDA [18]and ROC analyses were performed using R software (R Development Core Team [19]) with packages MASS[20] and ROCR[21], respectively, to automatically generate gene panels according to thefollowing algorithm (Figure 2):

1. The set of 'genes in model' is created. This set represents the genes that were not yet removed permanently. Initially, this set contains all genes. A set of genes with 'already tested genes' is also created. Initially, this set is empty.

2. The F-value, that is, the ratio of between-group variability and within-group variability, is calculatedfor each gene.

3. The classifier algorithm (LDA) is run using the set of 'genes in model'. The accuracy percentage value is recorded as the'best accuracy value'.

4. The set of 'selectable genes' is defined as:'selectable genes'= 'genes in model' minus 'already tested genes'. If the group of 'selectable genes' is not empty, the algorithm is continued in step 5. Otherwise, thealgorithm skips to step 7.

5. A gene is selected from the set of 'selectable genes' (to avoid performing the same calculation more than once as well as to terminate the algorithm) according to the following models:

a. Randomly with equal probabilities (uniform model);

b. Randomly with a probability that is inversely proportional to their F-value (F_propmodel);

c. Genes with the lowest F-values (min F model).

6. In either case, the selected gene is temporarily removed from the set of 'genes in model'.The advantage of using stochastic models instead of min F model is that theycan provide bettersegregation of patient groups. Uniform and F_prop models represent stochastic algorithms whilethe min F model is deterministic.

7. The classifier is run using the (temporarily reduced) set of 'genes in model'.

a. If the accuracy percentage value becomes lower, the selected gene isreinserted into the set of 'genes in model' and added to the set of 'already tested genes'.

b. If the accuracy percentage value is at least as good as the 'best accuracy value', theselected gene is permanently removed from 'genes in model' and the set of 'already tested genes'is emptied. The 'best accuracy value' is overwritten with the calculated accuracy value.The algorithm returns to step 4.

8. The algorithm ends. The outputs include the set of 'genes in model' and 'best accuracy value'.

## Results

### Clinical characteristics

In CD, we identified 14 respondersand 6 non-responders in the test cohort; and 13 responders and 7 non-responders in the validation cohort. There were no significant differences regarding age, CDAI, CRP, hemoglobin, leukocytes or neutrophils between the responders and non-responders (Table 1).

In RA, we used a binary outcome variable to assess clinical responder status: patients with ACR0% or ACR20% scores were classified as non-responders; and patients with ACR50% or ACR70% scores were classified as responders. We identified 6 responders and 13 non- or moderate-responders in the test cohort; and 4 responders and 11 non- or moderate-responders in the validation cohort. There were no significant differences regarding age, DAS28, HAQ, CRP, rheumatoid factor, anti-cyclic citrullinated peptide (anti-CCP)antibody status or DMARDs between respondersand non-responders (Table 2).

### Global gene expression analyses identify differentially expressed genes between responders and non-responders in Crohn's disease and rheumatoid arthritis

In CD, global gene expression analysis resulted in a list of 48 genes through filtering steps based on expression levels, fold-change cut-off at 1.5 and statistical significant analyses differentiating responders from non-responders at baseline. Analysis of samples obtained at week 2 identified 12differentially expressed genes with statistically significant differences between respondersand non-responders. Among these genes,*ABCC4*, *BMP6 *and *THEM5*were significantly changing at baseline as well; others were new findings at week 2, such as *CA2*, *CADM2*, *GPR34*, *IL1RL1*, *MMD*, *PRDM1*, *RAD23A *and *SLC7A5 *(Table S1a in Additional file 1).

In RA, analysis of baseline samples resulted in a list of 30 genes showing statistically significant differences between responders and non-responders. From this list, some of the genes such as *RGS1EPSTI1*, *IFI44*, *IFIT1*, *IFIT2*, *IFIT3*, *RFC1 *and *RSAD2 *were also significantly changed at week 2 as well, while others showed changes at week 2 only, such as *ELOVL7*, *FCGR3A*, *GPAM*, *MICA and PF4*(Table S1b in Additional file 1).There is no overlap between the final gene panels of CD and RA regarding the responder versus non-respondercomparison.

Comparing baseline and week 2 samples resulted in three genes (*AQP9*, *IGJ *and *TNFAIP6*) with statistically significant differences. These genes correlate with the effects of the therapy and disease progression over time.

There is no overlap between the final gene panels of CD and RA regarding the responder versus non-responder comparison, but *AQP9 *and *TNFAIP6 *overlapped regarding the baseline versus week 2 comparisons, which account for the effects of therapy.

### Biostatisticalanalysis of RT-quantitative PCR gene expression data

Genes that showed differential expression between responder and non-responder in the microarray experiment were validated on a biologically independent patient cohort in both conditions, using an RT-quantitative PCR method.

**Figure 3:**
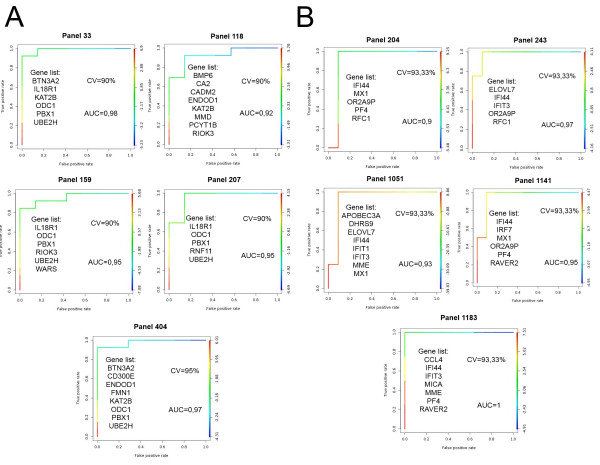
**Receiver operator characteristic-area under the curveanalyses**. The ratio between true and false positive discovery rates are shown in **(A) **Crohn's disease and **(B) **rheumatoid arthritis with the gene panels demonstrating an accuracy in differentiating between responder and non-responder patients of over 90%. CV stands for the success of cross-validation.

The RT-quantitative PCR data were analyzed with the LDA algorithm and lists of gene panels showing a perfect segregation between responder and non-responderwerecreated. Leave-one-out cross-validation was used to strengthen the statistical power of the lists.ROC-AUC analyses were visualized to show the true positive and false positive rates of gene panels with the bestcross-validation rates (Figure 3).

**Figure 4:**
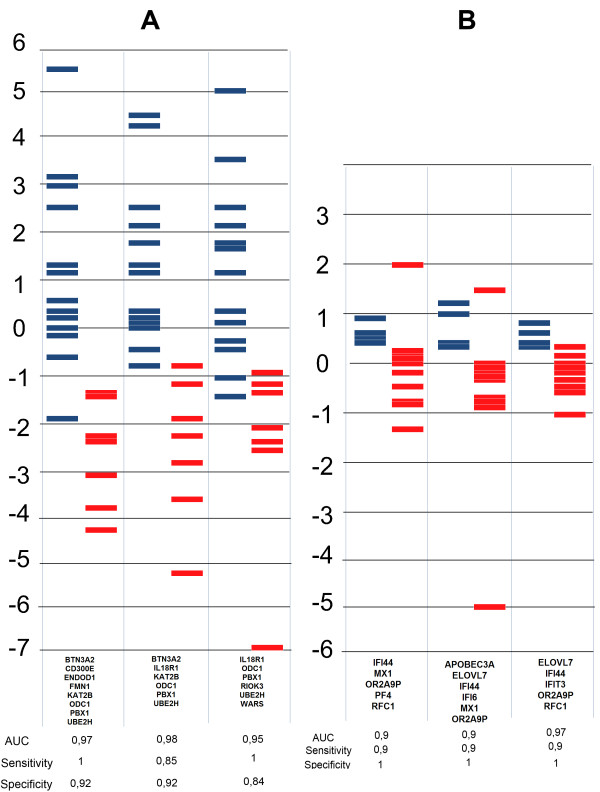
**Biostatistical analysis of gene expression data**. Three gene lists scored by linear discriminant analysisin **(A) **Crohn's disease and **(B) **rheumatoid arthritis. Red bars represent non-responders; blue bars represent responders. The larger the distance between the groups and the smaller the overlap between samples, the higher the power of separation of the gene list.

Based on the accuracy of cross-validation and the sensitivity of these gene panels, the threelists with the best discriminatory power were chosen for visualization in both conditions to show how responder and non-responder patients are segregated (Figure 4). The lists with the best discriminatory power include *BTN3A2*, *CD300E*, *ENDOD1*, *FMN1*, *KAT2B*, *ODC1*, *PBX1*and *UBE2H *in CD,and *IFI44*, *MX1*, *ORA2A9P*, *PF4*and *RFC1 *in RA.

Genes fromgene panels with cross-validation accuracy over 80% were ranked based on the number of times they appeared in such gene panels to show the most important genes in differentiating respondersfrom non-responders (Table 3).

**Table 3:** List of genes of gene panels with cross-validation accuracy over 80%

Crohn's disease		Rheumatoid arthritis	
**Number**	**Gene name**	**Number**	**Gene name**
59	*UBE2H*	26	*IFI44*
38	*ODC1*	19	*PF4*
33	*CD300E*	18	*RAVER2*
33	*PCYT1B*	16	*IFIT1*
31	*AIDA*	14	*IFIT3*
31	*RIOK3*	12	*APOBEC3A*
30	*PBX1*	12	*ELOVL7*
25	*ARHGEF12*	12	*MICA*
24	*MMD*	12	*OR2A9P*
23	*BMP6*	11	*IFI44L*
23	*WARS*	11	*MME*
22	*ENDOD1*	10	*CCL4*
21	*CYP1B1*	10	*RGS1*
21	*GCLC*	9	*IRF7*
20	*BTN3A2*	8	*EPSTI1*
20	*RNF11*	7	*MX1*
19	*CADM2*	6	*DHRS9*
19	*MAP1LC3B*	5	*RFC1*
17	*KAT2B*	5	*SERPING1*
16	*IL18R1*	3	*IFI6*
15	*FMN1*	2	*IFI35*
12	*CA2*	1	*IFITM1*
12	*IL1RL1*	0	*GZMB*

Order of genes based on the number of times included in gene panels with cross-validation accuracy over 80%. Number means the number of times the gene was included in such gene panels.

## Discussion

Predicting whether a patient responds to a specific biological therapy could have significant health and economic benefits, but a prediction based only on clinical parameters and disease activity scores does not yield the required efficacy. The detection of gene panels including genome classifiers discriminating between future responders and non-responders through the minimally invasive peripheral blood sampling either in CD or RA is clearly a yet unmet medical and diagnostic need. The most common approach to this problem is to identify individual genes showing statistically significant differences between responders and non-responders [10].

We combined these two approaches by performing global gene expression analyses in a test cohort to identify a panel of genes that later could be validated in an independent cohort. Our more sensitive method yielded several genes relevant to CD and RA based on the literature.

In CD, examples include *CYP1B1 *that has been marked as an inflammatory bowel disease marker compared to healthy controls in peripheral blood gene expression profiles [4]; *RNASE2 *was significantly reduced in patients with inflammatory bowel disease compared to healthy controls in peripheral polymorphonuclear leukocytes[22],as was *FCGR1A*, an inflammation-related gene thatis up-regulated in PBMCs of patients withulcerative colitis and CD [23]. A single nucleotide polymorphism of *IL18R1*[24] or *PRDM1*[25]was associated with CD.

As regulation of gene activity of interferon response during infliximab therapy in RA is associated with the treatment response based on whole blood gene expression profiling, it was not surprising to detect numerous genes related to the interferonpathways that had been previously investigated [10] such as *IFI44*, *IFI44L*, *IFIT1*, *IFIT2 *and *IRF2*. *PTGS2*also discriminated patients with RA from healthy controls in PBMCs at the gene expression level [4]; and the genetic polymorphism of *RFC1 *modifies methotrexate transport and metabolic effects and through that can influence response to treatment[26].

The RT-quantitative PCR technique was used to measure the expression levels of our pre-selected genes from the microarray analysis on biologically independent patient cohorts in both conditions; and LDA was applied to identify gene panels with the highest discriminatory power. Univariate analyses may disregard potential interactions among genes,but LDA can reveal underlying differences by using genes simultaneously as a gene panel, providing perfect segregation in the multidimensional space.

The high number of gene panels with 100% segregation and gene panels with accuracy of over 90% after cross-validation show that it is likely to find such panels when testing on larger cohorts. This could also mean that, regarding the development of a diagnostic assay predicting response to infliximab therapy in RA and CD, using a gene set containing 20 to 24 genes seems to be more reasonable than selecting individual gene lists consisting of typically 5 to 8 genes, provided that an LDA-based approach is used.

To provide compact gene panels resulting in a perfect segregation between responders and non-responders, as well as a success rate of over 90% after cross-validation, we detected and visualized these prominent gene panels - in which many of the genes overlapped in the different groups. Understanding the limitation of our study regarding the sizes of the cohorts, we created pool of genes which appeared the most times in the best performing gene panels to let other research groups test these in larger cohorts. A strategy combining such data sets and cohorts worldwide would have the highest chance of success in providing the community with validated gene lists usable in any cohorts.

This research could lead us to the diagnostic use of such gene arrays in predicting the response to infliximab therapy in CD and RA. The final conclusion of our study was that, althoughan ultimate gene panel might have been expected to be found, there is no such panel but instead a pool of genes with high statistical power that could be tested in further cohorts using LDA.

## Conclusions

In this work, we provided twopieces of proof of concept to show that peripheral blood gene expression profiles are suitable for determining gene panels with the highest discriminatory power that can differentiate responders from non-responders at baseline in CD and RA patient cohorts and can also be validated in independent cohorts; and despite the similar pathogenetic background of CD and RA, distinct, non-overlapping gene panels predict the responder status in these conditions.

Such gene panels couldcontribute to the solution of unmet needs in clinical decision making by determining in advance whether a patient willrespond to a specific and expensive biologic therapy by analyzing the gene expression patterns of the least invasively obtained peripheral blood samples, therefore prevent the patient from receiving an inefficient therapy and then cycling to an efficient one that could not then achieve the same efficacy as it would have done if used in the first place.

## Abbreviations

ACR: American College of Rheumatology; AUC: area under the curve; CD: Crohn's disease; CDAI: Crohn's disease activity score; CRP: C-reactive protein; CVA: canonical variates analysis; DAS28: Disease Activity Score for 28 Joints; DMARDs: disease-modifying anti-rheumatic drugs; HAQ: Health Assessment Questionnaire; LDA: linear discriminant analysis; PBMCs: peripheral blood mononuclear cells; RA: rheumatoid arthritis; ROC: receiver operator characteristic; TNFα: tumor necrosis factor alpha.

## Competing interests

ZsH is an employee of EGIS Nyrt. The remaining authors declare that they have no competing interests.

## Authors' contributions

BM designed the study, performed experiments and wrote the paper. SP designed the study and performed experiments. AV, KP, AV and ZSz performed sample collection and determined inclusion/exclusion criteria. GZ designed the study. AH and LS designed the biostatistical algorithm. JP performed multivariate analyses. LN directed research, designed the study and wrote the paper.All authors read and approved the final manuscript.

## Supplementary Material

Additional file 1 **Methods and Tables S1 and S2**. Description of canonical variates analysis. Table S1: Details of the genes used for validation. Table S2: Reasoning behind choosing ROC-AUC analysis. Click here for file
