# Supplementary material for: Peripheral blood derived gene panels predict response to infliximab in rheumatoid arthritis and Crohn's disease
Source: Genome Med. 2013 Jun 28;5(6):59. doi: 10.1186/gm463 (PMC4064310; doi:10.1186/gm463)
Supplement: Additional file 1 — Methods and Tables S1 and S2. Description of canonical variates analysis. Table S1: Details of the genes used for validation. Table S2: Reasoning behind choosing ROC-AUC analysis. [file gm463-S1.DOCX]

**Supplementary Materials**

**Supplementary Methods:**

**Canonical Variates Analysis**

Whereas PCA recovers underlying structures in the data without any a priori grouping of objects, separation between predefined groups of objects is best revealed by CVA. CVA was used to determine whether the groups of responders and nonresponders are separable in the multidimensional space spanned by the genetic variables, and if so, which gene subsets have the best discriminatory power. The results of CVA are the so-called canonical scores obtained from the canonical functions derived through eigenanalysis, which serve as coordinates of observations in the canonical space. Since the maximum number of canonical axes is 1 less than the number of groups, in our study CVA did not allow graphic display, and separation of responders and nonresponders is expressed merely by a list of scores for observations on a single canonical axis.

If the observations are taken at random and the variables satisfy multivariate normality, then statistical procedures are available to test the significance of group separation. Nevertheless, if these criteria are not met, as in our case, examination of the 2 groups as to whether they overlap on the canonical axis or not provides equally meaningful information. A partial limitation of CVA is that the number of variables cannot exceed the number of observations (patients). Therefore, many CVA runs were carried out using different subsets of genes, each subset defined on a logical basis. As a control, we used several sets of genes selected randomly from a set of genes known to have no influence on group separation.

Computations were performed using the Syn-Tax 2000 package.

**Supplementary Table 1: Details of the genes used for validation**

Lists of genes, which were chosen for validation based on our microarray results (baseline or week 2) and the related litreture. Genes marked with green are included in the gene panels with the best discriminatory power after the RT-QPCR validation.

**Supplementary Table 1a: List of the genes used for validation in Crohn’s disease**

|  |  |  |
| --- | --- | --- |
| Gene symbol | Description | Timepoint |
| *ABCC4* | ATP-binding cassette, sub-family C (CFTR/MRP), member 4 | Baseline + week 2 |
| *AIDA* | axin interactor, dorsalization associated | Baseline |
| *ARHGEF12* | Rho guanine nucleotide exchange factor (GEF) 12 | Baseline |
| *BMP6* | bone morphogenetic protein 6 | Baseline + week 2 |
| *BTN3A2* | butyrophilin, subfamily 3, member A2 | Baseline |
| *CA2* | carbonic anhydrase II | Week 2 |
| *CADM2* | cell adhesion molecule 2 | Week 2 |
| *CD300E* | CD300 antigen like family member E | Baseline |
| *CYP1B1* | cytochrome P450, family 1, subfamily B, polypeptide 1 | Baseline |
| *ENDOD1* | endonuclease domain containing 1 | Baseline |
| *FCGR1A* | Fc fragment of IgG, high affinity Ia, receptor (CD64) | Baseline |
| *FMN1* | formin 1 | Baseline |
| *GCLC* | glutamate-cysteine ligase, catalytic subunit | Baseline |
| *GPR34* | G protein-coupled receptor 34 | Week 2 |
| *HORMAD1* | HORMA domain containing 1 | Baseline |
| *IGF2BP2* | insulin-like growth factor 2 mRNA binding protein 2 | Baseline |
| *IL18R1* | interleukin 18 receptor 1 | Baseline |
| *IL1RL1* | interleukin 1 receptor-like 1 | Week 2 |
| *KAT2B* | K(lysine) acetyltransferase 2B | Baseline |
| *MAP1LC3B* | microtubule-associated protein 1 light chain 3 beta | Baseline |
| *MMD* | monocyte to macrophage differentiation-associated | Week 2 |
| *MS4A4A* | membrane-spanning 4-domains, subfamily A, member 4 | Baseline |
| *MS4A7* | membrane-spanning 4-domains, subfamily A, member 7 | Baseline |
| *ODC1* | ornithine decarboxylase 1 | Baseline |
| *PBX1* | pre-B-cell leukemia transcription factor 1 | Baseline |
| *PCYT1B* | phosphate cytidylyltransferase 1, choline, beta isoform | Baseline |
| *PIP4K2A* | phosphatidylinositol-5-phosphate 4-kinase, type II, alpha | Baseline |
| *PIP5K1B* | phosphatidylinositol-4-phosphate 5-kinase, type I, beta | Baseline |
| *PRDM1* | PR domain containing 1, with ZNF domain | Week 2 |
| *PSME4* | proteasome (prosome, macropain) activator subunit 4 | Baseline |
| *RAD23A* | RAD23 homolog A | Week 2 |
| *RIOK3* | RIO kinase 3 (yeast) | Baseline |
| *RNASE2* | ribonuclease, RNase A family, 2 (liver, eosinophil-derived neurotoxin) | Baseline |
| *RNF11* | ring finger protein 11 | Baseline |
| *SLC7A5* | solute carrier family 7 member 5 | Week 2 |
| *THEM5* | thioesterase superfamily member 5 | Baseline + week 2 |
| *TMEM176A* | transmembrane protein 176A | Baseline |
| *TMEM176B* | transmembrane protein 176B | Baseline |
| *UBE2H* | ubiquitin-conjugating enzyme E2H (UBC8 homolog, yeast) | Baseline |
| *WARS* | tryptophanyl-tRNA synthetase | Baseline |

**Supplementary Table 1b: Details of the genes used for validation in rheumatoid arthritis**

| Gene symbol | Description | Timepoint |
| --- | --- | --- |
| *APOBEC3A* | apolipoprotein B mRNA editing enzyme, catalytic polypeptide-like 3A | Baseline |
| *AQP9* | aquaporin 9 | Baseline vs Week 2 |
| *CCL4* | chemokine (C-C motif) ligand 4 | Sekiguchi et al |
| *CNTNAP3* | contactin associated protein-like 3 | Baseline |
| *CYP4F3* | cytochrome P450, family 4, subfamily F, polypeptide 3 | Baseline |
| *DHRS9* | dehydrogenase/reductase (SDR family) member 9 | Baseline |
| *EIF2AK2* | eukaryotic translation initiation factor 2-alpha kinase 2 | Sekiguchi et al |
| *ELOVL7* | ELOVL fatty acid elongase 7 | Week 2 |
| *EPSTI1* | epithelial stromal interaction 1 (breast) | Baseline + week 2 |
| *FCGR3A* | Fc fragment of IgG, low affinity IIIa, receptor (CD16a) | Week 2 |
| *GPAM* | glycerol-3-phosphate acyltransferase, mitochondrial | Week 2 |
| *GPR15* | G protein-coupled receptor 15 | Baseline |
| *GZMB* | granzyme B | Sekiguchi et al |
| *IFI35* | interferon-induced protein 35 | van Baarsen et al |
| *IFI44* | interferon-induced protein 44 | Baseline + week 2 |
| *IFI44L* | interferon-induced protein 44-like | Baseline |
| *IFI6* | interferon, alpha-inducible protein 6 | van Baarsen et al |
| *IFIT1* | interferon-induced protein with tetratricopeptide repeats 1 | Baseline + week 2 |
| *IFIT2* | interferon-induced protein with tetratricopeptide repeats 2 | Baseline + week 2 |
| *IFIT3* | interferon-induced protein with tetratricopeptide repeats 4 | Baseline + week 2 |
| *IFITM1* | interferon induced transmembrane protein 1 (9-27) | van Baarsen et al |
| *IL2RB* | interleukin 2 receptor, beta | Sekiguchi et al |
| *IRF2* | interferon regulatory factor 2 | van Baarsen et al |
| *IRF7* | interferon regulatory factor 7 | Sekiguchi et al |
| *MGAM* | maltase-glucoamylase (alpha-glucosidase) | Baseline |
| *MICA* | MHC class I polypeptide-related sequence A | Week 2 |
| *MME* | membrane metallo-endopeptidase | Baseline |
| *MX1* | myxovirus resistance 1, interferon-inducible protein p78 | Baseline |
| *OR2A9P* | olfactory receptor, family 2, subfamily A, member 9 pseudogene | Baseline |
| *PF4* | platelet factor 4 | Week 2 |
| *PTGS2* | prostaglandin-endoperoxide synthase 2 | Baseline |
| *RAVER2* | ribonucleoprotein, PTB-binding 2 | Baseline |
| *RFC1* | replication factor C (activator 1) 1, 145kDa | Baseline + week 2 |
| *RGS1* | regulator of G-protein signaling 1 | Week 2 |
| *RSAD2* | radical S-adenosyl methionine domain containing 2 | Baseline + week 2 |
| *S100P* | S100 calcium binding protein P | Baseline |
| *SERPINB10* | serine (or cysteine) proteinase inhibitor, clade B, member 10 | Baseline |
| *SERPING1* | serine (or cysteine) proteinase inhibitor, clade G, member 1, | Baseline |
| *SIGLEC1* | sialic acid binding Ig-like lectin 1, sialoadhesin | Baseline |
| *TNF* | tumor necrosis factor |  |
| *TNFAIP6* | tumor necrosis factor, alpha-induced protein 6 | Baseline vs Week 2 |

Genes whose expression levels could not be detected are listed here: *HLA DQ1, PRKR, OR2A20P|OR2A9P|OR2A5, MIR142, S100A8, S100A9, PTGES, IL11, IL6, G0S2, MST131* and *MIR1974|TOB2.*

**Supplementary Table 2**

We ran the leave-one-out validation, as you suggested, for all possible combinations and calculated the ROC AUC values for each gene panel. We got 15 different ROC AUC values for RA and 20 for Crohn’s disease. In case of several gene panels, all these values were equal to 1.

In the study, we chose a more conservative approach as we chose to run the leave-one-out validation but kept only the score of the actual test entity in order to get only one ROC AUC value from the different runs (per gene panel). The ROC AUC values were very high, even after the values were transformed to similar scale ((r-m)/s where r was the actual test entity score, m and s were the mean and standard deviation of the train and (one) test entities, respectively. As the scaling had no effect on the results, we kept the original (unscaled) ROC AUC values. These results were visualized on the ROC plots in the manuscript (Figure 3).

| Crohn ROC AUC values | | | | | |
| --- | --- | --- | --- | --- | --- |
| **LOOCV run** | **panel 404** | **panel 33** | **panel 118** | **panel 159** | **panel 207** |
| 1 | 1,0000 | 1,0000 | 1,0000 | 1,0000 | 1,0000 |
| 2 | 1,0000 | 1,0000 | 0,9560 | 1,0000 | 1,0000 |
| 3 | 1,0000 | 1,0000 | 1,0000 | 1,0000 | 0,9670 |
| 4 | 1,0000 | 1,0000 | 1,0000 | 1,0000 | 1,0000 |
| 5 | 1,0000 | 1,0000 | 1,0000 | 1,0000 | 1,0000 |
| 6 | 1,0000 | 1,0000 | 1,0000 | 1,0000 | 1,0000 |
| 7 | 1,0000 | 1,0000 | 1,0000 | 1,0000 | 1,0000 |
| 8 | 1,0000 | 1,0000 | 1,0000 | 1,0000 | 1,0000 |
| 9 | 1,0000 | 1,0000 | 1,0000 | 1,0000 | 1,0000 |
| 10 | 1,0000 | 1,0000 | 1,0000 | 1,0000 | 1,0000 |
| 11 | 1,0000 | 1,0000 | 1,0000 | 1,0000 | 1,0000 |
| 12 | 1,0000 | 1,0000 | 1,0000 | 1,0000 | 1,0000 |
| 13 | 1,0000 | 1,0000 | 1,0000 | 0,9890 | 1,0000 |
| 14 | 1,0000 | 1,0000 | 1,0000 | 1,0000 | 1,0000 |
| 15 | 1,0000 | 1,0000 | 1,0000 | 1,0000 | 1,0000 |
| 16 | 0,9890 | 1,0000 | 0,9670 | 1,0000 | 1,0000 |
| 17 | 1,0000 | 1,0000 | 1,0000 | 1,0000 | 1,0000 |
| 18 | 1,0000 | 1,0000 | 1,0000 | 1,0000 | 1,0000 |
| 19 | 1,0000 | 1,0000 | 1,0000 | 1,0000 | 1,0000 |
| 20 | 1,0000 | 1,0000 | 1,0000 | 1,0000 | 1,0000 |
| **Means** | 0,9995 | 1,0000 | 0,9962 | 0,9995 | 0,9984 |

| RA ROC AUC values | | | | | |
| --- | --- | --- | --- | --- | --- |
| **LOOCV run** | **panel 204** | **panel 243** | **panel 1051** | **panel 1041** | **panel 1183** |
| 1 | 1,0000 | 1,0000 | 1,0000 | 1,0000 | 1,0000 |
| 2 | 1,0000 | 1,0000 | 1,0000 | 1,0000 | 1,0000 |
| 3 | 1,0000 | 1,0000 | 1,0000 | 1,0000 | 1,0000 |
| 4 | 1,0000 | 1,0000 | 1,0000 | 1,0000 | 1,0000 |
| 5 | 1,0000 | 1,0000 | 1,0000 | 1,0000 | 1,0000 |
| 6 | 1,0000 | 1,0000 | 1,0000 | 1,0000 | 1,0000 |
| 7 | 1,0000 | 1,0000 | 1,0000 | 1,0000 | 1,0000 |
| 8 | 1,0000 | 1,0000 | 1,0000 | 1,0000 | 1,0000 |
| 9 | 1,0000 | 1,0000 | 1,0000 | 1,0000 | 1,0000 |
| 10 | 1,0000 | 1,0000 | 0,9545 | 1,0000 | 1,0000 |
| 11 | 1,0000 | 1,0000 | 1,0000 | 1,0000 | 1,0000 |
| 12 | 0,9090 | 1,0000 | 1,0000 | 1,0000 | 1,0000 |
| 13 | 1,0000 | 1,0000 | 1,0000 | 1,0000 | 1,0000 |
| 14 | 1,0000 | 1,0000 | 1,0000 | 1,0000 | 1,0000 |
| 15 | 1,0000 | 1,0000 | 1,0000 | 1,0000 | 1,0000 |
| **Means** | 0,9939 | 1,0000 | 0,9970 | 1,0000 | 1,0000 |
